# Supplementary material for: Buffering Capacity of Various Commercial and Homemade Foods in the Context of Gastric Canine Digestion
Source: Animals (Basel). 2023 Nov 27;13(23):3662. doi: 10.3390/ani13233662 (PMC10705345; doi:10.3390/ani13233662)
Supplement: Supplementary file 1 [file animals-13-03662-s001.zip › animals-2621869-supplementary.pdf]

Table S1. Ingredients of the dry and wet dog food as declared by the manufacturers.

ID	Ingredients
Wet food 1	Meat and animal derivatives (including 4% derivatives from beef), minerals, oils and fats, derivatives of vegetable origin, yeast (0,18%)
Wet food 2	Meat and animal by-products (lamb and chicken), vegetables (potato), minerals
Wet food 3	Meat and animal derivatives (50%, including 4% poultry), vegetables (including 4% mix of carrots and peas), grain (4% cooked rice), minerals, derivatives of vegetable origin
Wet food 4	57% meat and animal derivatives (including 30% from chicken and 4% from turkey), 4% fish and fish derivatives, grain (4% brown rice), minerals, oils and fats (0,8% salmon oil)
Wet food 5	Beef (21%), turkey, pork, dehydrated pork proteins, minerals, carrots (0,9% dehydrated carrots, corresponds to 8,1% carrots)
Wet food 6	Fresh turkey meat (42%), fresh pork meat (35%, Iberian pork only), fresh chicken meat (6%), salmon oil, dehydrated potato, dehydrated vegetables (broccoli, carrots, leek), dried brewers' yeast, olive oil
Wet food 7	32% salmon, 30% chicken (heart, liver, gizzard), stock form salmon, 29% chicken, 4% zucchini, 2% amaranth, 1% chia seeds, 1% krill, 0,5% minerals, 0,5 dried eggshells, 0,5% salmon oil
Wet food 8	68% lamb (heart, lung, liver, rumen), 2% amaranth, 0,8% cranberries, 0,2% salmon oil, calcium carbonate, sodium chloride
Wet food 9	Meat and animal derivatives (among other 10% venison), bakery products (5% cooked pasta), minerals, oils and fats (0,2% flaxseed oil), derivatives of vegetable origin (0,1% inulin)
Wet food 10	Meat and animal derivatives (51% including 21% beef, lamb and chicken), grain, minerals, derivatives of vegetable origin (among other 0,5% dried beet slices), oils and fats (among other 0,5% sunflower oil)
Dry food 1	Dehydrated poultry protein, maize, maize flour, animal fats, maize gluten, vegetable protein isolate, wheat, hydrolysed animal proteins, rice, beet

	pulp, minerals, fish oil, soya oil, yeasts and parts thereof, fructo-oligosaccharides
Dry food 2	Grain, meat and animal derivatives (4% from beef), derivatives of vegetables origin, oils and fats, sugar, vegetables (4% carrots), minerals
Dry food 3	Grain (wholemeal 57%), meat and animal derivatives (15%), derivatives of vegetable origin, oils and fats, vegetable protein extracts, glycerin, minerals, propylene glycol, dehydrated vegetables (0,25% peas and 0,25% carrots)
Dry food 4	Meat and animal derivatives 28% (4% veal), rice, maize, barley, millet, beef tallow, carrots 5%, chicory 2%, barm 1%, lignocellulose, flaxseed, blueberries 0,5%, seaweed, sodium chloride, dried dandelion, dried field horsetail, dried comfrey, dried borage, dried echinacea, dried rampion
Dry food 5	Cereals (maize, rice), meat and animal by-products (poultry meat meal, lamb meat meal), oils and fats, vegetable by-products (slivers of beet molasses), minerals, eggs and egg products (whole egg powder), yeast, algae (Ascophyllum nodosum), seeds (linseed), herbs, yeast extract (source of MOS), green-lipped mussel (Perna canaliculus), dried
Dry food 6	30% poultry meat, 16% maize, dried poultry protein, rice, 14% potato flour, 3% fishmeal, beet pulp, carob meal, dried barm, hydrolysed poultry liver, poultry fat, vegetable oil, dicalcium phosphate, sodium chloride, potassium chloride, dried herbage, Yucca schidigera
Dry food 7	Grain, meat and animal derivative (14% including 4% chicken), oils and fats (among other 0,4% sunflower oil), vegetable protein extracts, derivatives of vegetable origin, minerals, vegetables
Dry food 8	Fresh poultry (30 %), millet (20 %), maize, poultry protein (dried), dried beet pulp (sugar removed), rice, poultry fat, hydrolysed protein, linseed, powdered egg, fish meal, fish oil, peas (dried), yeast (dried, 0.1 % mannanoligo saccharides, 0.06 % beta-glucans), sodium chloride, green-lipped mussels (dried, 0.1 %), potassium chloride, chicory (dried)

Dry food 9	Fresh chicken meat (70 %), broken rice, corn (GMO-free), dehydrated chicken protein, hydrolysed poultry protein, dried brewers' yeast, dried apple pulp, salmon oil, yucca extract, cold-pressed flaxseed oil, cold-pressed olive oil, green-lipped mussel extract, dried carrots, dried tomatoes, dried african marigold, dried dandelion, dried broccoli, dried green tea, dried chamomile, dried oregano, dried milk thistle seed, dried cranberry seed, dried seaweed, potassium chloride
Dry food 10	Poultry protein, whole wheat, whole-grain maize, wheat flour, maize flour, whole-grain barley, lamb protein (8%), rice flour (8%), beet pulp (desugared), poultry fat, beef fat, hydrolysed liver, fish meal, sunflower oil (0.8%), apple pomace (0.8%), yeast, potassium chloride, rapeseed oil (0.2%), sodium chloride, green oats, sunflower, cress, parsley, green herbs (total: 0.3%) dried

Table S2. Ingredients of the homemade dog food.

ID	Ingredients
Homemade 1	60.3% potato, 36.2% codfish, 2.4% rapeseed oil, 1% vitaminized mineral feed, 0.1% sodium chloride
Homemade 2	52.6% green rumen, 44% potato, 2.1% rapeseed oil, 1.2% vitaminized mineral feed, 0.1% sodium chloride
Homemade 3	63.5% rice, 28.4% chicken, 5.4% carrots, 1.3% rapeseed oil, 1.3% vitaminized mineral feed, 0.1% sodium chloride
Homemade 4	59.3 rice, 21.2% chicken, 12.1% curd, 4.9% carrots, 1.2% rapeseed oil, 1.2% vitaminized mineral feed, 0.1% sodium chloride
Homemade 5	42.4% rice, 25.2% horse meat, 17.5% boiled egg, 12.1% carrots, 1.5% vitaminized mineral feed, 1.2% rapeseed oil, 0.1% sodium chloride
Homemade 6	43.4% rice, 41.3% horse meat, 12.4% carrots, 1.5% vitaminized mineral feed, 1.3% rapeseed oil, 0.1% sodium chloride
Homemade 7	53.1% oat flakes, 36.5% head meat from beef, 7.6% carrots, 1.5% vitaminized mineral food, 1.1% rapeseed oil, 0.2% sodium chloride

Homemade 8	56.2 oat flakes, 34.9% muscle meat from beef, 6.4% carrots 1.3% vitaminized mineral feed, 1.3% rapeseed oil, 0.2% sodium chloride
Homemade 9	45.2% pasta, 42% beef lung and heart, 10% carrots, 1.3% rapeseed oil, 1.3% vitaminized mineral feed, 0.2% sodium chloride
Homemade 10	61.5% pasta, 25.6% beef lung and heart, 10.3% carrots, 1.4% vitaminized mineral feed, 1% rapeseed oil, 0.2% sodium chloride

Tab. S3. Nutrient composition of each sample.

ID	DM content	Ash	CP	EE	ADF	NFC
		% of DM				
Wet food 1	18.33	10.40	43.34	24.05	11.30	10.91
Wet food 2	21.13	8.38	46.69	30.29	13.71	0.92
Wet food 3	17.07	10.91	48.88	15.31	15.16	9.75
Wet food 4	21.88	10.86	46.19	18.65	12.49	11.82
Wet food 5	22.49	10.82	52.87	18.81	20.10	0.00
Wet food 6	34.53	11.87	42.24	26.10	15.12	4.67
Wet food 7	26.25	6.62	35.36	19.85	19.80	18.36
Wet food 8	22.82	10.88	36.86	37.00	12.89	2.37
Wet food 9	25.62	11.86	42.41	29.58	21.31	0.00
Wet food 10	17.44	11.29	46.11	20.03	31.09	0.00
Dry food 1	90.74	5.67	26.25	17.63	16.96	33.49
Dry food 2	83.90	8.29	26.35	14.84	15.71	34.81
Dry food 3	88.49	9.28	25.46	10.40	12.41	42.45
Dry food 4	94.55	6.58	26.01	11.91	12.87	42.64
Dry food 5	92.61	6.57	27.68	14.05	11.38	40.31
Dry food 6	93.28	6.30	24.55	7.48	12.90	48.77
Dry food 7	92.93	7.07	23.21	10.91	14.28	44.52
Dry food 8	92.74	5.63	23.99	8.32	13.54	48.51
Dry food 9	83.64	8.82	33.56	17.22	19.82	20.60
Dry food 10	93.36	8.29	24.57	10.39	16.55	40.20
Homemade 1	21.76	7.67	36.51	9.46	10.12	36.24
Homemade 2	25.46	7.30	38.33	26.14	12.70	15.54
Homemade 3	26.15	8.18	24.69	11.98	9.34	45.81
Homemade 4	23.85	8.14	27.89	9.46	7.74	46.77
Homemade 5	30.41	5.43	24.33	32.32	7.39	30.53
Homemade 6	31.01	5.06	24.66	33.04	7.66	29.58

Homemade 7	29.04	6.27	27.19	24.81	10.08	31.65
Homemade 8	29.31	5.39	29.34	22.57	8.74	33.96
Homemade 9	19.19	5.11	32.88	12.41	11.07	38.53
Homemade 10	27.93	4.45	25.16	6.17	10.08	54.14

Table S4. Deviation of the measured vs. declared nutrients from manufactures in wet and dry dog food.[1]

	DM	CP	EE	Ash
Wet food				
Wet food 1	-3.5%	-18.9%	-19.8%	19.1%
Wet food 2	-18.7%	-10.3%	6.7%	10.7%
Wet food 3	-0.7%	-1.8%	-34.7%	-6.8%
Wet food 4	21.6%	1.1%	-18.4%	8.0%
Wet food 5	12.4%	13.2%	-24.4%	1.3%
Wet food 6	11.4%	0.6%	0.1%	63.9%
Wet food 7	19.3%	-15.6%	-19.8%	-13.1%
Wet food 8	-8.7%	-19.9%	5.5%	-0.7%
Wet food 9	16.4%	-1.2%	8.2%	21.5%
Wet food 10	-7.7%	4.5%	-36.5%	-21.2%
Average wet food	4.2%	-4.8%	-13.3%	8.3%
Dry food				
Dry food 1		-11.8%	0.0%	-4.8%
Dry food 2	4.9%	5.3%	3.7%	-4.7%
Dry food 3		7.3%	-8.0%	2.7%
Dry food 4		6.9%	2.3%	-11.2%
Dry food 5	1.8%	7.7%	6.7%	-10.5%
Dry food 6	3.6%	-0.4%	-33.6%	-16.1%
Dry food 7		2.7%	-22.0%	-12.4%
Dry food 8	0.8%	3.5%	-26.5%	-11.5%
Dry food 9	3.3%	8.0%	-10.0%	-6.7%
Dry food 10		4.3%	7.8%	19.0%
Average dry food	2.9%	3.3%	-7.9%	-5.6%

[1]Deviation=(measured-declared) x measured/100; Minus value indicates lower measured nutrient than declared in %, plus value indicates higher measured nutrient in %. The measured and declared nutrients were corrected by the respective DM content.

Tab. S5. Buffering capacity and used HCl of each sample.

ID	pH undiluted	Initial pH	BC	Used HCl	DM content	Sample amount DM	HCl/g DM	BC/g DM
				ml	%	g	ml/g DM	g/DM
Wet food 1	6.76	6.93	2.98	15.00	18.33	0.92	16.36	3.26
Wet food 2	.	6.56	2.77	13.00	21.13	1.06	12.30	2.62
Wet food 3	6.70	6.82	3.06	15.00	17.07	0.85	17.57	3.59
Wet food 4	6.87	6.89	3.22	16.00	21.88	1.09	14.62	2.94
Wet food 5	6.56	6.72	3.57	17.00	22.49	1.12	15.12	3.18
Wet food 6	6.14	6.28	4.17	18.00	34.53	1.73	10.43	2.42
Wet food 7	6.83	6.98	2.35	12.00	26.25	1.31	9.14	1.79
Wet food 8	6.45	6.64	2.54	12.00	22.82	1.14	10.52	2.23
Wet food 9	6.79	6.93	3.39	17.00	25.62	1.28	13.27	2.64
Wet food 10	6.84	6.98	2.18	11.00	17.44	0.87	12.61	2.50
Dry food 1	.	5.88	5.86	23.00	90.74	4.54	5.07	1.29
Dry food 2	.	5.57	10.92	40.00	83.90	4.20	9.54	2.60
Dry food 3	.	5.91	9.05	36.00	88.49	4.42	8.14	2.05
Dry food 4	.	5.47	9.08	32.00	94.55	4.73	6.77	1.92
Dry food 5	.	5.73	8.02	30.00	92.61	4.63	6.48	1.73
Dry food 6	.	5.51	7.89	28.00	93.28	4.66	6.00	1.69
Dry food 7	.	5.64	6.03	22.00	92.93	4.65	4.73	1.30
Dry food 8	.	5.39	6.38	22.00	92.74	4.64	4.74	1.38
Dry food 9	.	5.16	10.54	34.00	83.64	4.18	8.13	2.52
Dry food 10	.	5.91	8.44	34.00	93.36	4.67	7.28	1.81
Home made 1	.	6.79	2.49	12.00	21.76	1.09	11.03	2.29
Home made 2	6.43	6.55	2.80	13.00	25.46	1.27	10.21	2.20

Home made 3	6.12	6.37	2.28	10.00	26.15	1.31	7.65	1.74
Home made 4	5.16	5.81	2.57	10.00	23.85	1.19	8.38	2.15
Home made 5	6.14	6.35	2.28	10.00	30.41	1.52	6.58	1.50
Home made 6	5.84	6.05	2.65	11.00	31.01	1.55	7.09	1.71
Home made 7	5.97	6.24	2.57	11.00	29.04	1.45	7.58	1.77
Home made 8	6.00	6.22	2.32	10.00	29.31	1.47	6.82	1.58
Home made 9	6.11	6.40	2.21	10.00	19.19	0.96	10.42	2.31
Home made 10	6,09	6,34	183	8.00	27.93	1.40	5.73	1.31
